# Knowledge and awareness of the general public and perception of pharmacists about antibiotic resistance

**DOI:** 10.1186/s12889-018-5614-3

**Published:** 2018-06-08

**Authors:** Thuy Mason, Claire Trochez, Remmya Thomas, Maria Babar, Iman Hesso, Reem Kayyali

**Affiliations:** School of Life Sciences, Pharmacy and Chemistry, Kingston University London, Penrhyn Road, Kingston upon Thames, KT1 2EE UK

**Keywords:** Antibiotics usage, Antibiotic resistance, Pharmacists counselling, Public knowledge, Adherence

## Abstract

**Background:**

Antibiotic resistance (AR) continues to be a serious problem. Many factors contribute to AR, including inappropriate use of antibiotics, in which both healthcare professionals and patients play a contributing role. This study aimed to assess the awareness and knowledge of antibiotic usage and AR among the general public (in affluent and deprived areas) and community pharmacists’ (CPs’) in Greater London.

**Methods:**

A cross-sectional survey involving members of the public was conducted between July 2014 and February 2015. Stage one involved members of the public (*N* = 384) residing in affluent areas of London. The second stage targeted public (N = 384) in deprived areas of London. In addition, CPs (*N* = 240) across the same areas were also surveyed. Data analysis was performed using Microsoft Excel and SPSS Software packages.

**Results:**

Response rate: 36% (*n* = 139/384) and 57% (*n* = 220/384) and 25% (*n* = 60/240) of public residing in affluent areas, deprived areas and of CPs respectively was achieved. Definitive trends in knowledge of how antibiotics work could not be drawn to distinguish between affluent and deprived areas. However, public respondents residing in affluent areas possessed better understanding of AR and prudent use of antibiotics, and this was statistically significant in both cases (*p* < 0.05). Exposure to an antibiotic campaign (32% in affluent areas, 17% in deprived areas) did not raise public respondents’ knowledge on AR and only partially raised their general knowledge on antibiotics usage. Only 20% of public residing in deprived areas received counselling from a CP, among them 74% had an antibiotic prescribed on at least one previous occasion. Those who received counselling displayed better knowledge about concordance/adherence with respect to antibiotic usage (*p* < 0.05) whereas exposure to an antibiotic campaign made no significant impact on knowledge about concordance/adherence.

**Conclusion:**

The study highlights that there has been no change in the status quo with respect to awareness of antibiotic usage and AR even after the implementation of several awareness campaigns in England. Those who benefited from CP counselling showed a significant better knowledge towards prudent antibiotic usage which stresses the importance of CPs’ counselling on antibiotic prescription.

## Background

Alexander Fleming, the man who discovered penicillin in 1928, and who subsequently received his Nobel Prize in 1945 for this work, predicted in his then winning speech that the world would one day be facing antibiotic resistance (AR) [1–5]. Indeed his prediction has now become a reality [2], as the whole world is heading towards a “post-antibiotic era” [6].

Several factors play a contributing role in the development of AR. While inappropriate use of antibiotics has been identified as the main cause behind AR [7, 8]; there are still other causes for it including the use of antibiotics in the food production industry and in animals’ health making them key reservoirs of antibiotic resistant bacteria [9, 10].

Both healthcare professionals (HCPs) and patients are responsible for AR; there is a direct correlation between use and overuse of antibiotics and the emergence of AR [7, 11, 12]. In the case of HCPs, it is most likely due to inappropriate prescribing. In the case of patients, it is most likely because of overusing or not taking a full course of treatment; or self-medication by sharing medication with other people, or keeping part of the course for another occasion or acquisition from pharmacies without a prescription [8, 13]. Therefore, ensuring appropriate use of antibiotics is crucial to enable resistance to be reduced [8, 14]. In Europe, the tremendous burden of AR was estimated to be €1.5 billion annually incorporating healthcare costs and productivity losses [15–18]. In the UK, more than 40 million prescriptions are issued for antibiotics every year, costing the National Health Service (NHS) £192 m [19). In return, another research within the UK highlighted that 1 in 4 antibiotic prescriptions are unnecessarily prescribed, equating to a total of 10 million unnecessary prescriptions each year [19].

The threat of AR is increasing at an alarming rate [14], making simple infections untreatable or routine medical procedures almost impossible in the near future [20]. The magnitude of the threat of AR has been reported to be comparable to that of climate change [21]. AR has been widely acknowledged as a global public health issue and a global challenge [6, 9, 12, 14, 19], that leads to increased healthcare costs, rise in new adverse reactions to antibiotics and increased mortality [6, 8]. Estimates indicate an average death toll of 25,000 people each year in Europe due to antibiotic-resistant bacteria [16–18, 21]. This highlights the need for an urgent response from every corner of the globe to strengthen measures to combat AR [21–23].

AR would not be such a great concern if there were a continuous supply of new novel antibiotics. Since pharmaceutical companies have not been forthcoming with development of any new antibiotics with novel mechanisms of action in recent years [24–27], efforts have been made to reduce the rate and stem the extent of AR by raising awareness in the community at large and targeting the public specifically.

Global collaboration to encourage the prudent use of antibiotics and raise awareness of AR has been promoted through awareness-raising activities/ campaigns in Australia, USA,Canada and most European countries [14, 28]. In 1999, the Department of Health in England had attempted to invest in campaigns like ‘*Andybiotic – Don’t wear me out’* [29] in which the awareness was raised. A survey conducted in 2003 revealed that awareness of the campaign was low and 43% (*p* < 0.0005) of the people still believed that antibiotics work on viruses [30]. Campaigns such as the European Antibiotic Awareness Day (EAAD) introduced in 2008, focused on educating the general public about AR and promoting the sensible use of antibiotics as well as taking measures to reduce the risk of infections e.g. maintaining good hygiene [28]. The campaign also encouraged prescribers to prescribe wisely, providing tools like a checklist that can be used to optimise antibiotic prescribing [31]. McNulty et al. [32] compared attitudes towards antibiotics usage of members of the public before (*n* = 1888) and after (*n* = 1830) the EAAD campaign to evaluate its success. No improvement was seen in the respondents’ understanding of correct antibiotics usage. Furthermore, the results detected a significant rise in the number of respondents keeping leftover antibiotics and thus casting doubt over the evidence that the campaign was effective [32].

This demonstrates the fact that one campaign at a point in time does not sufficiently promote the awareness amongst the general public. There is a need for continual public awareness campaigns covering large percentages of the public. As part of the EAAD, in September 2014, the Antibiotic Guardian Campaign was launched in England where all members of the society, including HCPs are asked to make a pledge that encourages the individual to take on measures that combat antimicrobial resistance [33].

HCPs such as community pharmacists (CPs) are in the best position to help in reducing AR due to their level of antibiotic knowledge and accessibility to the community [34, 35]. This can be done by providing counselling to patients about antibiotics prescriptions, particularly dosage intervals, side effects and interactions with other medications or food, given that 80% of antibiotics are prescribed in primary care [29, 36]. In England, CPs are expected to provide counselling about medications as part of their contractual framework through essential services such as dispensing to ensure patients’ safe use of their medicines [37]. In addition, the minor ailment scheme in England has been commissioned through CPs as part of their contractual framework as an enhanced service to aid general practitioners (GPs) with winter pressures [38]. The scheme has been introduced so that CPs can provide advice and support about the management of minor ailments including coughs, colds, sore throats and earaches [39], through which antibiotics can be potentially prescribed unnecessarily, without the need to visit the GP practice [39].

This study aimed to assess the awareness and knowledge of antibiotic usage and AR among the general public and to assess CPs’ knowledge about AR in London. The study was conducted among public residing in affluent and deprived areas, with a hypothesis that such a socio-demographic factor can be related to awareness and knowledge of AR, with public in affluent areas having more awareness and knowledge of AR, since the incidence of infectious diseases tends to be higher in groups with lower socioeconomic status and outcomes tend to be poorer [40].

## Methods

This is a cross-sectional survey study that was conducted in two stages. Stage one involved members of the public in affluent areas, whereas stage two involved members of the public in deprived areas, in addition to CPs in both affluent and deprived areas.

### Public survey

Members of the public were approached in person by two researchers in affluent public areas of London (Wimbledon, Richmond and Kingston upon Thames), and another two researchers in deprived public areas or in areas with diverse ethnicities (Hackney, Waltham Forest, City of Westminster, Haringey, Newham, Islington, Enfield). Affluent and deprived areas were determined based on the latest published index of multiple deprivations (IMD) score [41] and were chosen due to convenience based on proximity to the researchers but the distribution of the questionnaires was randomized.

A sample size of 384 members of the public in affluent areas and 384 in deprived areas were approached at random in public places and handed the questionnaire. The total adult population in the included boroughs was 327,078 and 1,196,845 for affluent and deprived areas respectively. The sample size calculation was done using Raosoft sample size calculator [42] providing a confidence level of 95% with margin of error of 5%, which indicated the need to approach 384 in affluent and deprived areas. Members of the public were included in the study if they were: between 18 and 65 years, able to read and understand English to enable them complete the questionnaire and willing to participate. Those who consented to participate filled out and handed the questionnaire back to the researchers. Data was collected between July 2014 and February 2015.

The questionnaire was structured to facilitate self-administration by the public, and was partially based on a previous survey related to the topic [43] and aimed to collect data about public attitudes, knowledge and awareness levels of AR, as well as exposure to an antibiotic awareness campaign through TV, radio, GP surgeries or pharmacies. The second stage survey for the public residing in deprived areas had an additional question about whether they received counselling about antibiotic usage once prescribed to them. Both surveys consisted mostly of close-ended questions, some Likert scale questions and multiple response questions with one or two open-ended questions at the end to enable participants the freedom of expression in answering .

### CP survey

240 Pharmacies were selected by convenience sampling in the same areas through which public were approached. The sample size was also calculated using the Raosoft sample size calculator [42]. The total number of pharmacies in the included boroughs in the study is 612 (32 Kingston, 82 Sutton and Merton including Wimbledon, 45 Richmond, 94 Westminster, 63 Waltham forest,65 Hackney, 57 Haringey, 68 Newham, 45 Islingston, 61 Enfield) [44]. Therefore, the total sample size calculated for the included boroughs was 237 at a 95% confidence level and 5% margin of error.

CPs were sent a postal survey with a prepaid envelope for returning the questionnaire in January 2015. The questionnaire was posted with an information sheet detailing the aims of the study, maintenance of confidentiality and the importance of participation. Only one response was received from each pharmacy. To maximise response rate, a follow-up questionnaire package was sent to CPs who didn’t provide an initial response, after one month in February 2015. Only fully completed questionnaires that were returned up until end of February 2015 were considered for the final analysis.

The questionnaire for this phase was designed by the researchers and focused on the role CPs played in increasing awareness about AR and whether they feel there is a need to do more for the community in this regard.

### Pilot study

A pilot study was done to validate both CPs and public questionnaires after obtaining ethical approval and prior data collection.

For CP survey, the pilot phase aimed to test content and face validity of the questionnaire used. The researchers discussed the questionnaire topics informally with 5 CPs in South London. Afterwards, the CPs were asked to fill the questionnaire and provide any comments about the questions on the chosen topics. The content was deemed satisfactory by the CPs; however, minor changes were required, which involved changing the wording of some questions.

Whereas the public survey was piloted on a sample of 30 members of the public in South London to test for face and content validity. People were asked to fill the questionnaire, to indicate whether the questions were clear in relation to the topic and to provide any comments to improve the questionnaire. Minor amendments were required which involved changing the wording of some questions in order to facilitate self-administration. The results obtained in the pilot study were not included in the final analysis to avoid any type of bias.

### Data analysis

All responses were coded before analysis. Simple statistical analyses (Chi square test) were employed to determine whether a relationship exists between responses to questions on knowledge, behaviour and respondents’ demographics. A *p* value of < 0.05 was considered to be statistically significant.

### Ethical consideration

Ethical approval for both stages of the study was obtained from the Delegated Research Ethics Committee at Kingston University London (Ref: 1213/045).

## Results

A total of 384 members of the public were approached in affluent areas and 139 agreed to participate giving a response rate of 36%. Whereas, in deprived areas, 384 were approached and 220 agreed to participate, giving a response rate of 57%. As for CPs, 240 questionnaires were mailed; however only 60 completed the questionnaire, providing a response rate of 25%.

### General knowledge on antibiotics usage and on antibiotics resistance possessed by respondents in affluent and deprived areas

Table 1 shows that definitive trends in knowledge of how antibiotics work cannot be drawn to distinguish between affluent and deprived areas. 81% (*n* = 112/139) of respondents in affluent areas displayed better understanding by disagreeing with the statement *‘antibiotics help cure the common cold, cough and flu’* compared with 45% (*n* = 98/220) in deprived areas (*p* < 0.05). Whereas, 68% (*n* = 149/220) of respondents in deprived areas displayed better understanding by disagreeing with the statement *‘antibiotics are effective against fungi’* compared with 43% (60/139) in affluent areas (p < 0.05). Both sets of respondents possessed comparatively poor knowledge as only 55% (*n* = 77/139) in affluent areas and 46% (*n* = 101/220) in deprived areas knew that antibiotics are not effective against virus.

**Table 1:** Awareness of respondents in affluent and deprived areas on correct antibiotic usage

STATEMENTS *(correct response)*	Affluent Areas % (n) % = n/N (*N* = 139)	Deprived Areas % (n) % = n/N *(N = 220)*	*x* ^*2*^ *p*
Antibiotics help cure the common cold, cough and flu *(disagree)*	81 (112)	45 (98)	*x*^*2*^ *= 45.5467* *p < 0.05*
Antibiotics are effective against fungi *(disagree)*	43 (60)	68 (149)	*x*^*2*^ *= 21.1259* *p < 0.05*
Antibiotics are effective against viruses *(disagree)*	55 (77)	46 (101)	x^2^ = 3.0666 *p* = 0.079918
Antibiotics are effective against bacteria *(agree)*	80 (111)	78 (171)	*x*^*2*^ *= 0.2291* *p =* 0.632171

As for knowledge on issues surrounding AR, Table 2 shows that respondents in affluent areas exhibited a much better understanding on the subject, where 81% (*n* = 112/139) correctly agreed that *‘antibiotic resistance is when antibiotics no longer work to treat infections’*, compared to 57% (*n* = 126/220) in deprived areas, (*p* < 0.05) and 88% (*n* = 123/139) agreed that *‘if taken too often or unnecessarily, antibiotics are less likely to work in the future’*, compared to 61% (*n* = 135/220) in deprived areas, (*p* < 0.05).

**Table 2:** Respondents’ knowledge surrounding antibiotic resistance (AR) in affluent and deprived areas

STATEMENTS *(correct response)*	Affluent Areas % (n) % = n/N (N = 139)	Deprived Areas % (n) % = n/N *(N = 220)*	*x* ^*2*^ *p*
Antibiotic resistance is when antibiotics no longer work to treat infections *(agree)*	81 (112)	57 (126)	x^2^ = 20.7008 *p* < 0.05
If taken too often or unnecessarily, antibiotics are less likely to work in the future *(agree)*	88 (123)	*61 (135)*	x^2^ = 30.9991 *p* < 0.05

### Knowledge on prudent antibiotics usage of respondents in affluent and deprived areas

Respondents in affluent areas showed a much better understanding on prudent antibiotics usage compared to those in deprived areas (Table 3), with 99% always *‘taking antibiotics as prescribed’* and 85% never *‘stopped taking antibiotics when symptoms have improved’*, compared to 58% (*n* = 95/163) and 34% (*n* = 55/163) respectively in deprived areas (p < 0.05).

**Table 3:** Knowledge on prudent antibiotics usage of respondents in affluent and deprived areas

STATEMENTS *(correct response)*	Affluent Areas % (n) % = n/N (*N* = 134)	Deprived Areas % (n) % = n/N *(N = 163)*	*x* ^*2*^ *p*
Following instructions/taking antibiotics as prescribed is important *(always)*	99 (133)	58 (95)	x^2^ = 69.2196 *p* < 0.05
Stop taking antibiotics when symptoms have improved *(never)*	85 (114)	34 (55)	x^2^ = 79.0194 *p* < 0.05

### Demographic factors, counselling and campaign exposure impact on the knowledge and behaviour of respondents in deprived areas

The responses provided by respondents in deprived areas were further analysed.

Ethnicity and language did not influence their general knowledge on antibiotic usage whereas education (at a degree level or above), being in a healthcare profession or having a family member working in healthcare partly enhanced but not always significantly, their general knowledge on antibiotic usage (Table 4).

**Table 4:** Influence of ethnicity, language, education level, profession and campaign exposure on antibiotic knowledge in deprived areas

Statements on antibiotic usage *(correct response)*	Ethnicity % (n) % = n/N		Language % (n) % = n/N		Education % (n) % = n/N		Profession % (n) % = n/N		Profession of family member % (n) % = n/N		Campaign exposure % (n) % = n/N	
	White N = 36	Non-white *N* = 184	English as first language *N* = 94	English not first language N = 126	Below degree level *N* = 125	Degree level or above N = 95	Health-care related *N* = 44	Not health-care related *N* = 176	Health-care related *N* = 75	Not health-care related *N* = 145	Yes N = 37	No N = 183
Antibiotics are effective against fungi *(no)*	75 (27)	67 (123)	69 (65)	67 (85)	66 (83)	71 (67)	77 (34)	66 (116)	73 (55)	66 (95)	59 (22)	70 (128)
*×* ^*2*^ *p*	*0.9224* *0.336856*		*0.0708* *0.790232*		*0.4236* *0.515129*		*2.0952* *0 .147759*		*1.392* *0.238066*		*1.5599* *0.21168*	
Antibiotics are effective against viruses *(no)*	47 (17)	46 (84)	40 (38)	50 (63)	37 (46)	58 (55)	73 (32)	39 (69)	61 (46)	38 (55)	54 (20)	44 (81)
*x* ^*2*^ *p*	*0.0299* *0.862744*		*1.98740* *0.158615*		*9.6724* *0.001871*		*15.9294* *0.000066*		*10.9019* *0.000961*		*1.1883* *0.275671*	
Antibiotics are effective against bacteria *(yes)*	78 (28)	78 (143)	82 (77)	75 (94)	71 (89)	86 (82)	93 (41)	74 (130)	85 (64)	74 (107)	84 (31)	77 (140)
*x* ^*2*^ *p*	*0.0001* *0.993646*		*1.6625* *0.197263*		*7.124* *0.007606*		*7.588* *0.005876*		*3.8027* *0.051171*		*0.9425* *0.331641*	
Antibiotics can treat sore throat *(strongly disagree/disagree)*	33 (12)	41 (76)	43 (40)	38 (48)	34 (43)	47 (45)	59 (26)	35 (62)	47 (35)	37 (53)	57 (21)	37 (67)
*x* ^*2*^ *p*	*0.7971* *0.371961*		*0.4458* *0.504338*		*3.7825* *0.051793*		*8.3523* *0 .003852.*		*2.1073* *0.1466*		*5.2041* *0.022534*	
Antibiotics help cure the common cold, cough and flu *(strongly disagree/disagree)*	36 (13)	46 (85)	43 (40)	46 (58)	34 (43)	58 (55)	70 (31)	38 (67)	59 (44)	37 (54)	59 (22)	42 (76)
*x* ^*2*^ *p*	1.2396 *0*.265555		*0.2637* *0.60758*		*12.0618* *0.000515*		*14.9461* *0 .000111*		*9.1859* *0.002439*		*4.0052* *0.045361*	
Antibiotics can speed up recovery of a cold/flu *(strongly disagree/disagree)*	31 (11)	36 (66)	31 (29)	38 (48)	30 (37)	42 (40)	59 (26)	29 (51)	52 (39)	26 (38)	43 (16)	33 (61)
*x* ^*2*^ *p*	*0.3737* *0.540977*		*1.2419* *0.265113*		*3.7104* *0.054076*		*14.031* *0 .00018*		*14.4555* *0.000144*		*1.3286* *0.249058*	

In affluent areas, only 32% (*n* = 45/139) of respondents have been exposed to an antibiotic awareness campaign compared to an even lower 17% (*n* = 37/220) of respondents in deprived areas. Exposure to an antibiotic awareness campaigns in deprived areas partially raised public respondents’ general knowledge on antibiotics usage but not on AR (Tables 4 and 5a).

**Table 5:** Impact of counselling and antibiotic awareness campaign on AR and concordance/adherence in deprived areas

**a Respondents’ knowledge on Antibiotic Resistance**				
STATEMENTS *(correct response)*	CAMPAIGN EXPOSURE % (n) % = n/N			
	Yes N = 37	No N = 183		
Bacteria has potential to become resistant to antibiotics *(strongly agree/agree)*	73 (27)	69 (126)		
*x* ^*2*^ *p*	*0.2467* *0.619391*			
Antibiotic resistance is when antibiotics no longer work to treat infections *(strongly agree/agree)*	54 (20)	63 (115)		
*x* ^*2*^ *p*	*1.0024* *0.316725*			
If taken too often or unnecessarily, antibiotics are less likely to work in the future *(strongly agree/agree)*	62 (23)	56 (103)		
*x* ^*2*^ *p*	*0.4345* *0.509766*			
**b Concordance/adherence behaviour of respondents in deprived areas who have had an antibiotic prescribed on at least one previous occasion**				
STATEMENTS *(correct response)*	Campaign exposure % (n) % = n/N		Counselling exposure % (n) % = n/N	
	Yes *N* = 33	No *N* = 130	Always N = 32	Not always/Sometimes/never *N* = 131
Do you follow the instructions given to take an antibiotic? *(always)*	64 (21)	57 (74)	84 (27)	52 (68)
*x* ^*2*^ *p*	*0.4878* *0.48489*		*11.1494* *0.000841*	
Do you complete the full course of antibiotics prescribed? *(always)*	45 (15)	46 (60)	75 (24)	39 (51)
*x* ^*2*^ *p*	*0.0052* *0.942617*		*13.4687* *0.000243*	
Do you stop taking the antibiotics once you start feeling better? *(never)*	27 (9)	35 (46)	59 (19)	27 (36)
*x* ^*2*^ *p*	*0.7746* *0.378784*		*11.7015* *0.000624*	
Do you skip doses of antibiotics? (never)	33 (11)	33 (43)	56 (18)	27 (36)
*x* ^*2*^ *p*	*0.0008* *0.977704*		*9.6082* *0.001937*	

The responses of public in deprived areas who have had an antibiotic prescribed to them on at least one previous occasion (*n* = 163/220) were further analysed to investigate the impact of counselling and antibiotic campaigns on their antibiotic consumption behaviour (Table 5b). However, only 20% (*n* = 32/163) stated that they always received counselling on antibiotic usage. Respondents who received counselling on antibiotic usage displayed better knowledge about concordance/adherence behaviour with respect to antibiotic usage than those who did not always receive counselling (*p* < 0.05) (Table 5b). Exposure to an antibiotic campaign made no significant impact on respondents’ knowledge about concordance/adherence behaviour with respect to antibiotic usage (Table 5b).

### Response on whether antibiotics had been obtained by participants without a prescription

In affluent areas, 96% (*n* = 134/139) participants recalled that they have had an antibiotic on at least one previous occasion, 92% (*n* = 123/134) confirmed the antibiotics were prescribed to them on a prescription. However, 5% (*n* = 7/134) of respondents admitted they had some antibiotics left over from a previous supply and 1% (n = 1/134) indicated the supply was purchased over the counter (OTC). None of the respondents indicated that friends/family had given them antibiotics, however 3% (*n* = 4/134) specifically requested their doctor to prescribe an antibiotic but were refused.

In deprived areas, 74% (*n* = 163/220) of respondents recalled that they have had antibiotics on at least one previous occasion. 90% (*n* = 146/163) confirmed the antibiotics were prescribed to them on a prescription. However, some participants also admitted that they had obtained antibiotics from various other means. Notably 12% (*n* = 19/163) from family/friends, 7% (*n* = 12/163) from abroad, 9% (n = 14/163) from a ‘previous supply’, and 7% (*n* = 11/163) from the internet.

Interestingly, 75% (*n* = 45/60) of pharmacist respondents indicated they had been requested to supply an antibiotic without a prescription. The main requests for antibiotics were for cold/flu (64%, *n* = 29/45) followed by for sore throat (24%, n = 11/45).

### Relationship of the sampled general public and their prescriber

In affluent areas, 73% (*n* = 101/139) of participants indicated they would never insist on having an antibiotic prescribed to them. However, only 33% (*n* = 46/139) said they would trust their doctor if an antibiotic was ever refused to be prescribed to them. Whereas, in deprived areas, 66% (*n* = 108/163) of participants who had an antibiotic on at least one previous occasion indicated they hadn’t insisted on having an antibiotic prescribed to them. When asked whether they would trust their doctor even if they did not prescribe them an antibiotic, although 36% (58/163) remained neutral, 21% (35/163) indicated that they would strongly distrust their doctor if an antibiotic is not prescribed.

### Level of engagement of CPs in raising AR awareness amongst the general public

80% (*n* = 48/60) of CPs admitted to not ever initiate a campaign to raise awareness on AR although 81% (*n* = 49/60) agreed with the importance of running the campaigns. The reported low uptake of pharmacist respondents in running the antibiotic awareness campaign was due to several factors. Notably lack of engagement was cited to be due to lack of funding (60%, *n* = 36/60), high workload/time constraints (58%, *n* = 35/60), followed by language barrier with patients/communication problems (53%, *n* = 32/60).

## Discussion

The current findings shed light on the existing situation pertaining to antibiotic usage and awareness of AR among the public in affluent and deprived areas of London.

Overall, public respondents residing in affluent areas possessed much better knowledge and understanding on AR problems and antibiotic usage and this was statistically significant (*p* < 0.05), which in return supports our hypothesis. In addition, public respondents in deprived areas possessed much poorer knowledge on prudent antibiotic usage compared to those in affluent areas (p < 0.05). In the current study, 88% in affluent areas agreed that antibiotics become ineffective if used unnecessarily compared to 61% in deprived areas. In this regard, a recent report by the European commission demonstrated that knowledge of antibiotics among the public has remained constant since 2013 with 84% of participants knew that antibiotics would become ineffective if used unnecessarily [45]. Nevertheless, the current results are also comparable to those reported by McNulty et al. [29], 10 years ago, which in return still highlights the lack of improvement in public knowledge about AR and the need to improve the status quo.

Although public respondents in both affluent and deprived areas were equally knowledgeable (80 and 78% respectively) that antibiotics are effective against bacteria, they could not differentiate between bacteria and viruses and hence, believed that antibiotics would work against diseases caused by viruses also. Our results showed that 45 and 54% in affluent and deprived areas respectively believed that antibiotics are effective against viruses. Unfortunately, this is again comparable to the results of the survey study that was conducted in Britain10 years ago by McNulty et al. [29] after the Andybiotic campaigns in 1998 and 2002, where 43% (3062/7120) of population believed that antibiotics can kill viruses. This in return calls into question the success of the antibiotic awareness campaigns as it highlights that any efforts to date to educate the general public have not made a remarkable impact on this front. In the current study, exposure to the antibiotic awareness campaigns did not enrich knowledge of those respondents on how to take an antibiotic correctly, as the responses did not differ statistically to those who had not been exposed to campaigns.

In deprived areas, 55% of respondents incorrectly believed that antibiotics can treat sore throat, cure common cold, cough and flu; 34% also admitted that they had insisted that an antibiotic be prescribed to them. In addition, CPs in the current study indicated that cold/flu and sore throat were the main conditions for which people request antibiotics from a pharmacy without prescription. Patients demand antibiotics perhaps because they often misunderstand the antibiotic action against microbes and the different models of prudent usage. In a UK study, 55% of 1000 GPs surveyed admitted that they felt under pressure from patients demanding an antibiotic. Importantly, 44% revealed that although they felt an antibiotic was neither appropriate nor necessary, they prescribed it anyway to close the consultation with the patient [46]. In another survey in England, 5 out of 6 GPs reported to feel pressured to prescribe an antibiotic when it is not necessary [38]. Physicians are more likely to prescribe antibiotics under pressure in order to meet the patient’s expectation and satisfaction [38, 46, 47]. In our survey, 67% of respondents in affluent areas and 21% in deprived areas indicated that they would distrust their doctor if their request for an antibiotic is refused.

Considering that 80% of prescribed antibiotics occur in primary care [29, 36, 48], CPs can play a potential role in counselling their patients [38]. In the current study, participants in deprived areas who received counselling displayed a statistically significant better understanding on how to correctly take an antibiotic, so it is disappointing to see that under 20% (*n* = 32/163) in deprived areas actually received counselling on antibiotic usage from a CP, given that counselling on safe use of medicines is a part of the dispensing service which is provided by all pharmacy contractors with NHS England [37]. On the other hand, this highlights the importance of CPs in this field and comes in line with previous calls in England for CPs to become “*antibiotic guardians*” as one way to halt the spread of AR [38].

In the current study, although 81% of CPs indicated that AR awareness campaigns are important to educate members of the public, yet their motivation in running such campaigns is lacking, with 80% not ever initiating an antibiotic awareness campaign. This was reflected with the small percentages of the public who were exposed to an antibiotics awareness campaign (32 and 17% in affluent and deprived areas respectively). However, this highlights/suggests lack of CPs’ efforts for curtailing the global crisis of AR, and stresses the importance to have joint efforts by all HCPs and members of the public to address the issue of AR [38].

A small percentage of respondents admitted to taking antibiotics that were not prescribed for them by a qualified HCP. The revelation by some respondents that they have leftover antibiotics (5% in affluent areas and 9% in deprived areas) was similar to the 10% of those surveyed in other studies [49, 50]. In our survey, this inappropriate behaviour is marginally less yet struck a chord with that conducted by Pechère et al. [51] which reported that the public held inaccurate belief about the appropriate usage of antibiotics with 62% keeping leftover antibiotics for future use. Pechere [52] noted that patients frequently report discontinuing antibiotic therapy when they begin to feel better which might be the very reason why they would have leftover antibiotics. The implication of keeping leftover antibiotics, besides being an indicator of poor compliance with antibiotic therapy [53], has been widely accepted as being a contributing factor to AR [8, 53], but the potential three-fold impact on AR has not yet been fully realised. Firstly, stopping the antibiotic too soon is likely to allow bacteria to proliferate leading to AR. Secondly, the left over antibiotics may not be suitable for the next infection and thirdly, even if they were suitable, the amount left over is unlikely to be sufficient for the desired treatment duration [53]. This depicts the need for creating public awareness that antibiotics should only be used with a valid prescription and demands appropriate counselling on finishing the course as prescribed to ensure that the effectiveness of antibiotics is preserved [2, 53]. Although, this latter matter is currently under debate [54].

Communication between HCPs and their patients should play an important part in conveying correct usage of antibiotics to reduce AR. Horne [55] highlighted the importance of patient-provider interactions and communication so that HCPs can efficiently support patient informed choice to promote optimal adherence both individually and as part of a multidisciplinary team. Yet in this study, 53% of CPs cited that language barrier was a factor hindering them communicating with their patients.

### Study limitations

The current study has several limitations that should be taken into consideration when interpreting the findings. The study was conducted only within some regions in London which limits the generalisability of the results on a national scale. Furthermore, the effect of counselling on knowledge regarding antibiotic behaviour was only studied from the responses acquired from the public in deprived areas of London. The sample size of those responses was rather small, thus limiting the generalisation of the influence of demographic factors, campaign exposure and counselling on antibiotic knowledge and adherence. On the other hand, although two attempts were made to boost response rate for CPs’ survey, yet the response rate obtained was small to be generalizable. In general, studies employing a self-administered questionnaire have other potential limitations including non-response bias, recall bias and social desirability bias due to the nature of the data being self-reported [43, 56]. The cross-sectional nature of the study also highlights that the data is collected at one point in time [43]. Despite these limitations, the current findings provide update about the public’s knowledge of antibiotic usage and awareness of AR. Given the global recognition and threat of AR, a similar study on a national scale in the UK among the public and CPs is recommended to provide a more comprehensive and profound insight into the existing status pertaining to antibiotic usage and awareness of AR.

## Conclusion

The public surveyed still had deficiencies in antibiotic knowledge, which highlights that there has been no change in the status quo with respect to awareness of antibiotic usage and AR even after the implementation of several awareness campaigns in England, although respondents from affluent areas performed better overall compared to those in deprived areas. On the other hand, those who benefited from CP counselling showed a significant better knowledge towards prudent antibiotic usage which highlights the importance of CPs’ counselling on every antibiotic prescription. In an era where AR is a ticking time bomb, stringent measures in prescribing and dispensing antibiotics should be followed, with a view to making pharmacist counselling on every antibiotic prescription compulsory.

## Abbreviations

AR: Antibiotic resistance
CP: Community pharmacist
EAAD: European Antibiotic Awareness Day
GP: General practitioner
HCPs: Healthcare professionals
IMD: Index of multiple deprivations
NHS: National Health Service
OTC: Over the counter
